# Prenatal assessment of high-risk pregnancies in primary and specialized outpatient care: a mixed study

**DOI:** 10.1590/0034-7167-2022-0420

**Published:** 2023-11-10

**Authors:** Fabiana Fontana Medeiros, Izabel Dayana de Lemos Santos, Juliana Vicente de Oliveira Franchi, Sebastião Caldeira, Rosângela Aparecida Pimenta Ferrari, Sandra Marisa Pelloso, Maria do Carmo Fernandez Lourenço Haddad, Alexandrina Aparecida Maciel Cardelli

**Affiliations:** I Universidade Estadual de Londrina. Londrina, Paraná, Brazil; II Universidade Estadual do Oeste do Paraná. Cascavel, Paraná, Brazil; III Universidade Estadual de Maringá. Maringá, Paraná, Brazil

**Keywords:** Prenatal Care, Pregnancy, High-Risk, Primary Health Care, Secondary Care, Health Services Administration, Atención Prenatal, Embarazo de Alto Riesgo, Atención Primaria de Salud, Atención Secundaria de Salud, Administración de los Servicios de Salud, Pré-Natal, Gestação de Alto Risco, Atenção Primária, Atenção Secundária à Saúde, Gestão dos Serviços de Saúde

## Abstract

**Objectives:**

to assess high-risk prenatal care and identify strategies for improving care.

**Methods:**

a mixed study of a prospective cohort, with 319 mothers in a public maternity hospital, from October 2016 to August 2017, using a semi-structured instrument and interview. Analysis was performed using the chi-square test (p≤0.05). The qualitative approach was carried out through interviews with guiding questions to 13 managers, at their workplace, between January and March 2020, analyzed under social phenomenology.

**Results:**

higher rates of inadequacy were identified for all criteria. However, when care was shared, there was a higher rate for performing tests (p=0.023), consultations (p=0.002), risk stratification (p=0.013) and emergency information (≤0.000). Weaknesses in the record evidenced impairment in communication and continuity of care.

**Final Considerations:**

shared care is a strategy for improving care, however there is a need to strengthen effective referral and counter-referral to care continuity.

## RESUMO

**Objetivos:**

avaliar a assistência pré-natal de alto risco e identificar as estratégias para o aperfeiçoamento assistencial.

**Métodos:**

estudo misto de uma coorte prospectiva, com 319 puérperas em uma maternidade pública, de outubro de 2016 a agosto de 2017, utilizando-se instrumento semiestruturado e entrevista. Realizou-se análise por meio do Teste do Qui-Quadrado (p≤0,05). A abordagem qualitativa foi realizada por entrevista com questões norteadoras a 13 gestores, em seu local de trabalho, entre janeiro e março de 2020, analisadas sob a fenomenologia social.

**Resultados:**

identificaram-se maiores taxas de inadequação para todos critérios. Entretanto, quando a assistência foi compartilhada, observou-se maior taxa para realização de exames (p=0,023), consultas (p=0,002), classificação de risco (p=0,013) e informação de emergência (≤0,000). Fragilidades no registro evidenciaram prejuízo na comunicação e na continuidade assistencial.

**Considerações Finais:**

o cuidado compartilhado demonstra-se estratégia ao aperfeiçoamento assistencial, entretanto há necessidade do fortalecimento da referência e contrarreferência efetivas à continuidade assistencial.

## RESUMEN

**Objetivos:**

evaluar la atención prenatal de alto riesgo e identificar estrategias para mejorar la atención.

**Métodos:**

estudio mixto de cohorte prospectivo, con 319 madres en una maternidad pública, de octubre de 2016 a agosto de 2017, mediante instrumento semiestructurado y entrevista. El análisis se realizó mediante la prueba de chi-cuadrado (p≤0,05). El abordaje cualitativo se realizó a través de entrevistas con preguntas orientadoras a 13 directivos, en su lugar de trabajo, entre enero y marzo de 2020, analizados bajo la fenomenología social.

**Resultados:**

se identificaron mayores tasas de inadecuación para todos los criterios. Sin embargo, cuando se compartió la asistencia, hubo mayor tasa de realización de pruebas (p=0,023), consultas (p=0,002), clasificación de riesgo (p=0,013) e información de emergencia (≤0,000). Las debilidades en el expediente evidenciaron afectación en la comunicación y continuidad de la atención.

**Consideraciones Finales:**

el cuidado compartido es una estrategia para mejorar la atención, sin embargo, existe la necesidad de fortalecer la referencia y la contrarreferencia efectivas para la continuidad del cuidado.

## INTRODUCTION

Prenatal (PN) care aims at the gestational development, the childbirth of a healthy newborn and the absence of harm to maternal health, adding a psychosocial approach and educational activities^(1)^. In Brazil, PN care has achieved wide coverage^(2)^.

Maternal and child health policies have promoted access to safe and quality health care^(3)^. Despite the advances achieved, maternal mortality remains a major public health problem in the world^(4)^. Complications related to the gestational period, childbirth and the puerperium are the main causes of death and disability in women of reproductive age^(5)^.

In this context, even though access to PN care is universal, care inadequacy is observed when the minimum recommendations recommended by the Program for Humanization in Prenatal and Birth (PHPN - *Programa de Humanização no Pré-natal e Nascimento*) and the Mother and Child Care Network (RAMI - *Rede de Atenção Materna e Infantil*) are jointly analyzed^(2, 6, 7)^.

Therefore, it is important to ensure, in a timely manner, effective care for pregnant women at gestational risk as well as access to high-risk (HR) outpatient services, seeking to minimize the consequences for maternal and neonatal health^(8)^.

There is a gap in the literature regarding studies that investigate the weaknesses and potential of care in PN care^(9)^. The pertinence of studies that assess PN care in a comprehensive way, according to recommended criteria, through public health policies, seeking improvements in high-risk prenatal (HRPN) care is highlighted.

## OBJECTIVES

To assess HRPN care and identify strategies for improving assistance.

## METHODS

### Ethical aspects

The study was approved by the Research Ethics Committee at the *Universidade Estadual de Londrina*.

### Study design, period and site

This is a mixed study with a sequential explanatory design, with a quantitative-qualitative approach, defined by the quantitative data having greater attribution of weight, being collected and analyzed in a first step of the research “QUAN”^(10)^. For this 1st phase, data from a prospective cohort study, carried out in a public maternity hospital in southern Brazil, a reference for HR childbirth, from October 2016 to August 2017, was used. analysis of qualitative data with less weight “qual”, performed on the quantitative results^(10)^. Phase 2 took place, through a phenomenological study, having as a scenario participants’ own workplace, namely: the Municipal Health Department; the State Secretariat of the 17th Regional Health; the Specialty Clinic of a public university; and the Middle Paranapanema Intermunicipal Health Consortium. All were located in southern Brazil, and occurred between January and March 2020.

### Sample, and inclusion and exclusion criteria

In the QUAN step, a sample calculation of 1,447 consultations in 2015 was considered, with a sampling error margin of 5% and a confidence level of 95%, defining a number of 319 postpartum women. In this phase, women admitted to the maternity hospital under study and who were in the immediate puerperium and without cognitive impairment were included. Exclusion criteria included women who were hospitalized to undergo a procedure or just for gestational treatment.

In the qual phase, intentional sampling was used, defining as inclusion criteria managers who worked in indirect gestational HR care, in Primary Health Care (PHC) and in Specialized Outpatient Care (SOC). A minimum time of 60 days in the management position was also considered. The number of participants was not defined, *a priori*. Inclusion criteria were considered for the composition of subjects and the inclusion of managers from all institutions surveyed. Six subjects were excluded from the study, of which 4 did not meet the inclusion criteria and 2 did not accept to participate in the study, thus having 13 participants, who were identified by the letter G (manager), followed by the numbering from 1 to 13.

### Study protocol – quantitative step

Socioeconomic, demographic, obstetric and puerperal data were used as independent variables, and as a dependent variable, shared care (being this PN care performed in PHC concomitantly with SOC and non-shared care (when PN care is performed only in the PHC or only in the SOC).

To record the information, a semi-structured instrument was used to compile the records. As a guarantee of information quality and data collection instrument adequacy, a pilot test was carried out with six postpartum women, which it was observed that the instrument did not have specific topics related to cesarian surgery, and the necessary adaptations were made to the instrument, inserting more information in the case of cesarian section. It should be noted that the six postpartum women were excluded from the final sample.

The study followed the following steps: identification of women according to inclusion criteria; consultation and transcription of records in prenatal booklet (PNB), hospital records; post-childbirth interview during hospitalization; and two telephone contacts on the 10^th^ post-childbirth day and after the 42^nd^ post-childbirth day. Data were collected daily until the composition of the sample. Nineteen women were excluded, for whom it was not possible to be contacted by telephone or interviewed at home after hospital discharge, and it was not possible to continue the interview in the post-childbirth period.

Data collection was carried out by the researcher with *stricto sensu* training, together with a group of undergraduate, *lato sensu* and *stricto sensu* graduate students, who were previously trained.

### Study protocol – qualitative step

First, managers involved in indirect care management in HRPN care were identified, through the administrative team, through telephone contact. Subsequently, an interview was scheduled with the manager, according to participants’ availability.

Data were collected through individual interviews using a voice recorder, using an instrument as a guide, through two questions: how do you perceive PN care for HR pregnant women? What do you believe could be implemented in this care? The description of participants relied on a semi-structured instrument. An interview was carried out as a pilot test, with no need to change the instrument. Thus, the interview was considered in the sample, as there was no need to carry out repeated interviews. The interviews were carried out only with the presence of the participant and the researcher, and had an average duration of 50 minutes. The interviews were closed as soon as the contents of the reports were repeated and the proposed objective was achieved.

Data collection was carried out by the main author of the study, who at the time had a master’s degree in obstetrics and a professional bond as a nursing professor. The researcher had experience in qualitative study and deepening of the approached method.

The transcripts were returned to participants for comments and editing of information, being returned to the researcher later.

### Analysis of results, and statistics – quantitative step

After data collection (QUAN), a descriptive and analytical analysis of PN care adequacy was carried out using the Statistical Package for Social Sciences (SPSS), version 20.0. Three theoretical frameworks were used to classify HRPN care adequacy: World Health Organization (WHO) recommendations on PN care regarding nutritional interventions (advice on diet, physical activity and daily oral supplementation of ferrous sulfate and folic acid during pregnancy); maternal and fetal assessment (regarding laboratory tests); preventive measures (related to immunization); and interventions in health systems to improve utilization and quality of care (referring to the pregnant woman having BNP and the occurrence of the first PN consultation up to the 12^th^ week of gestational age)^(11)^.

The *Rede Mãe Paranaense* Guide Line for 2012 and 2018, adding to preventive measures (exams for uterine and oral health cancer prevention, and laboratory tests, for diagnosis of urinary tract infection and maternal-fetal blood compatibility), added to maternal and fetal assessment (laboratory tests and ultrasound),integrating interventions in health systems to improve utilization and quality of care (number of PN consultations, gestational risk stratification, sharing care with SOC, participation in a pregnant women’s group, bonding and visiting the maternity, and puerperal follow-up and reproductive)^(3, 12)^. The Technical Note for the Health Care Network (RAS – *Rede de Atenção à Saúde*) organization focusing on PHC and SOC elected the care markers (physical examination of pregnant women and identification of warning signs), adding to preventive measures (immunization and culture of group B *streptococcus* - GBS) and adding to interventions in health systems to improve utilization and quality of care (second puerperal consultation)^(13)^.

The aforementioned guidelines were adapted, electing cut-off points for classifying PN care adequacy, considering the possibility of carrying it out according to the gestational period and the indispensability of carrying out care at any time during PN care, classifying assistance as adequate or inadequate (Chart 1).

**Chart 1:** Proposal of criteria for assessing prenatal quality, Londrina, Paraná, Brazil, 2022

**Nutritional interventions**	
**Adequate** (present all components)	**Inadequate** (present one of the components)
Guidance on nutrition	Not receiving any or only one guidance associated with not supplementing with iron and/or folic acid
Guidance on physical exercise	
Daily oral iron and folic acid supplementation	No use of supplementation
**Maternal and fetal assessment**	
**Adequate** (present all components)	**Inadequate** (present one of the components)
Blood glucose test (three trimesters)	No trimester
Hemoglobin and hematocrit test (two or more records)	No record
Hemoglobin electrophoresis test (1^st^ trimester)	No trimester
HIV serology*(1^st^ and 3^rd^ or all trimesters)	None or only one trimester
Serology for VDRL† (all trimesters)	None or only one trimester
HBsAg exam‡ (1^st^ trimester)	No trimester
Serology for toxoplasmosis (susceptible pregnant woman all trimesters) and (non-susceptible 1^st^ trimester)	None or only one trimester in a susceptible pregnant woman
Three ultrasound scans, the first one before the 24^th^ week GA§ and the second between the 26^th^ and 28^th^ week GA§	None or one record
**Preventive measures**	
Adequate (present all components)	**Inadequate** (present one of the components)
Group B Streptococcus culture at 35^th^ to 37^th^ week GA§	No record
Urinalysis I and urine culture (all trimesters)	None or just one trimester
ABO blood screening|| and Rh¶ (1^st^ trimester)	No record
Pap smear (1^st^ trimester) or in the period ↓ one year of gestation	Late for ≥ one year of the gestational period
Tetanus vaccine (dTpa** during pregnancy)	No records or schemes
Hepatitis B vaccine (three doses or vaccine up to date)	No records or schemes
Influenza vaccine (one dose in the current pregnancy)	No records in current pregnancy
≥ one dental consultation during pregnancy	No dental consultation
**Health system interventions to improve utilization and quality of PN care**	
Adequate (present all components)	**Inadequate** (present one of the components)
Having a PN booklet	Not having a PN booklet
Start PN before GA§12^th^ week	Starting PN after GA§ 12^th^ week
≥ seven PN consultations	< seven consultations PN
Risk rating record (all consultations)	No record
Informing which hospital to go to in an emergency	Not receiving the information
PN performed at PHC concomitant with SOC	Not performing PN or performing only at PHC or only at SOC
Maternity visit during PN care	-^††^
Participation in ≥ a group of pregnant women	-^††^
Origin of the pregnant woman to carry out the childbirth in the same city or transport carried out by ambulance or medical transport	-^††^
Puerperal follow-up in the 1^st^ week and 30-40 days post-childbirth	No care
Reproductive method indicated by physician or nurse	-^††^
**Care markers in PN follow-up**	
**Adequate** (present all components)	**Inadequate** (present one of the components)
≥ six GA^§^ records	Having zero-two record(s)
≥ one LPD^‡‡^ and PCD^§§^ record (calculate in all consultations)	Not having any registration
≥ six blood pressure records	Having zero-two record(s)
≥ six maternal weight records	Having zero-two record(s)
≥ six fetal heartbeat records	Having zero-two record(s)
≥ six uterine height records	Having zero-two record(s)
≥ three fetal status and presentation records	Having zero-two record(s)
≥ one change record detected in PN care	Not having any record

*HIV – Human Immunodeficiency Virus; †VDRL – Venereal Disease Research Laboratory; ‡HBsAg – Hepatitis B virus surface antigen; §GA – gestational age; ||ABO – classification of human blood; ¶Rh – blood group system; **dTpa – adult-type acellular bacterial triple; ††− – failure to provide care classifies care as intermediate; ‡‡LPD – last period date; §§PCD – probable childbirth date.

After analyzing HRPN care adequacy, gaps were identified in PN follow-up, analyzed from weaknesses in the care received and insufficient records and guidelines given, in addition to not sharing care with SOC with all pregnant women, there is a need to deepen the phenomenon “care management for HR pregnant women”. Thus, the study was followed up in the second step (qual).

### Theoretical-methodological framework and data analysis – qualitative step

In the second phase of this study (qual), the methodological theoretical framework of Alfred Schütz’s social phenomenology was chosen^(14)^, through understanding the meaning of HRPN care management as well as the reasons for professional action in care management and actions that can be implemented to improve care. Data organization and analysis was guided by means of: accurate reading of each report, identifying the units of meaning, from participants’ lived experience; performing the grouping of significant aspects for composing the categories; analysis of categories, listing the “reasons why” of actions already experienced and the “reasons for”, represented by future actions expressed in the reports, resulting in the categorization of data and the understanding of the phenomenon in the light of Alfred Schütz’s social phenomenology^(15)^.

Participants’ reports emerged in a context of meanings, experienced so far considered “reasons why” as well as the perspectives presented in the “reasons for” in relation to management actions in HR gestational care. Two concrete categories of experience were identified, described as “reasons why”: *Experiencing the difficulty in communication between services*; and the category concerning perspectives: *Strategies to be carried out for articulation between services*, described as “reasons for”.

In the presentation of testimonies, there was correction in the transcription of the Portuguese edited to the cultured norm, not changing the meaning of participants’ speeches.

## RESULTS

The first step of this study (QUAN) had 319 postpartum women, most of whom were in the reproductive age range of 20-35 years (70.5%) and extremes of age (29.5%). As for race, the largest portion self-declared white (57%), with a partner (86.8%) and high school (55.8%). Almost half of the sample had a family income greater than three minimum wages (49.3%), 35.3%, between two and three minimum wages, and 15.4%, up to one minimum wage (current minimum wage: R$980.00, or US$196.00, Brazil, 2017).

With regard to obstetric data, 38.2% were primiparous, 32.9% were second-parous, and 28.8% were multiparous, and the minority had an interpartum interval of less than two years (12.2%). With regard to the current mode of childbirth, there was a predominance of cesarian section (60.2%) and gestational age for childbirth above 37 weeks (74.3%).

The description of HRPN care adequacy is presented in Table 1.

**Table 1:** Distribution of adequacy of high-risk prenatal care regarding nutritional interventions, maternal and fetal assessment, preventive measures, improvements in utilization and quality of care and markers of care according to the place where high-risk prenatal care is performed, Londrina, Paraná, Brazil, 2022

Variables	PN performance place						Total	
	Primary Care		Secondary Care		Primary and Secondary Care			
	n	%	n	%	n	%	n	%
Nutritional interventions								
Adequate	8	13.3	2	5.7	27	12.1	37	11.6
Intermediary	16	26.7	15	42.8	94	42.1	125	39.3
Inadequate	37	60.0	18	51.5	102	45.8	157	49.1
Maternal and fetal assessment								
Adequate	5	8.3	7	20.0	44	19.7	56	17.6
Intermediary	5	8.3	6	17.1	25	11.2	36	11.3
Inadequate	51	83.4	22	62.9	154	69.1	227	71.1
Preventive measures								
Adequate	3	5.0	4	11.4	28	12.5	35	11.0
Intermediary	-	-	-	-	-	-	-	-
Inadequate	58	95.0	31	88.6	195	87.5	284	89.0
Improvements in utilization and quality of care^†^								
Adequate	-	-	-	-	1	0.5	1	0.3
Intermediary	3	5.3	-	-	83	39.3	86	28.7
Inadequate	53	94.7	33	100.0	127	60.2	213	71.0
Care markers								
Adequate	4	6.7	7	20.0	48	21.5	59	18.5
Intermediary	2	3.3	3	8.6	8	3.6	13	4.1
Inadequate	55	90.0	25	71.4	167	74.9	247	77.4
Total	61	100.0	35	100.0	223	100.0	319	100.0

^†^ *Considered a sample of 300 postpartum women (excluding 18 women who could not be contacted in the final quantitative phase).*

**Figure 1:**
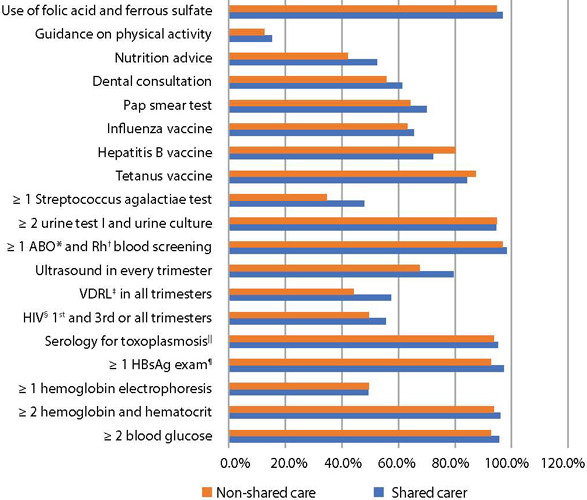
Distribution of nutritional interventions, preventive measures, maternal and fetal assessment, according to type of care received, Londrina, Paraná, Brazil, 2022

Nutritional interventions, maternal/fetal assessment and preventive measures are described in Figure 1.

*ABO – classification of human blood; †Rh – blood group system; ‡VDRL – Venereal Disease Research Laboratory; §HIV – Human Immunodeficiency Virus; ||Examination performed in the 1^st^, 2^nd^, 3^rd^ trimesters in susceptible pregnant women or 1^st^ trimester in non-susceptible pregnant women; ¶HBsAg – hepatitis B virus surface antigen.

There was a statistically significant difference between HRPN care adequacy, as measured through maternal and fetal assessment, and improvements in utilization and quality of care, and care markers for the type of care provided. Likewise, improvements in utilization and quality of care were more evident in the group of postpartum women monitored in shared care, except for the waiting time to start the outpatient follow-up, which was longer than 15 days. As for care markers, shared care also stood out. The lack of record of the situation and fetal presentation was relevant (Table 2).

**Table 2:** Distribution of high-risk prenatal adequacy regarding nutritional interventions, maternal and fetal assessment, preventive measures, improvements in utilization/quality of care and care markers according to type of care provided during high-risk prenatal care, Londrina, Paraná, Brazil, 2022

Variables	Type of care performed						
	Shared care		Non-shared care		Total		*p* value*
	n	%	n	%	n	%	
Nutritional interventions							
Receiving guidance on nutrition	117	52.5	40	42.1	157	49.3	0.090
Receiving guidance on physical activity	34	15.2	12	12.6	46	14.5	0.540
Maternal and fetal assessment							
VDRL^†^ trimester							
1^st^ + 2^nd^ + 3rd trimesters	128	57.4	42	44.2	170	53.5	0.023
Only 2 trimesters	56	25.1	28	29.5	84	26.4	
Ultrasound performance trimester							
1^st^ + 2^nd^ + 3rd trimesters	177	79.4	64	67.4	241	75.8	0.020
Only 2 trimesters	28	12.5	17	17.9	45	14.1	
Improvements in utilization and quality of care							
≥ 6 PN^‡^ consultation records	211	94.6	80	84.2	291	91.5	0.002
Having a risk stratification record	169	75.8	59	62.1	228	71.7	0.013
Participation in a pregnant group	64	28.7	13	13.7	77	24.2	0.003
Know which service to seek in an emergency	174	78.0	56	58.9	230	72.3	≤0.000
Know the reason for referral to HRPN care^§^	206	92.4	59	62.1	265	83.3	≤0.000
Waiting to start outpatient follow-up^||^							
Up to 7 days	45	20.2	6	6.3	51	16.0	≤0.000
8-15 days	39	17.5	12	12.6	51	16.0	
>15 days	139	62.3	17	17.9	156	49.1	
Did not access the SOC	-	-	61	63.2	61	18.9	
Care markers							
Record of the problem identified in PN^‡^ care	132	59.2	45	47.4	177	55.7	0.052
≥ 6 blood pressure records	209	93.8	79	83.1	288	90.6	0.007
≥ 6 maternal weight records	210	94.2	76	80.0	286	89.9	≤0.000
≥ 6 gestational age records	192	86.1	68	71.6	260	81.8	0.001
3-5 gestational age records	25	11.2	18	18.9	43	13.5	
≥ 6 fetal heartbeat records	192	86.1	52	54.8	244	76.8	≤0.000
3-5 fetal heartbeat records	24	10.8	33	34.7	57	17.9	
≥ 6 uterine height records	195	87.5	53	55.8	248	78.0	≤0.000
3-5 uterine height records	21	9.4	31	32.6	52	16.3	
3-5 fetal status and presentation records	101	45.3	27	28.4	128	40.2	0.004
Total	223	100.0	95	100.0	318	100.0	

*Obtained by the Chi-Square Test (p<0.05); †VDRL – Venereal Disease Research Laboratory; ‡PN – prenatal care; §HRPN CARE – high-risk antenatal care; ||Included by the author as a recommendation for interventions to improve utilization and quality of care; SOC – specialized outpatient care.

The second step of this study (qual) had 13 managers, 5 of whom belonged to the Municipal Department of Health, in southern Brazil, (4) to the State Department of Health of the 17^th^Health Region and (4) to the SOC. As for the employment relationship, there was a predominance of public servants (10). Most managers were women (11), aged between 36 and 62 years. Regarding professional training, there was a predominance of nursing (9), and the others were medicine, pharmacy and foreign languages (4). The average training time was 22 years. All participants had *lato sensu* (9) and *stricto sensu* (4) graduate degrees.

**Figure 2:**
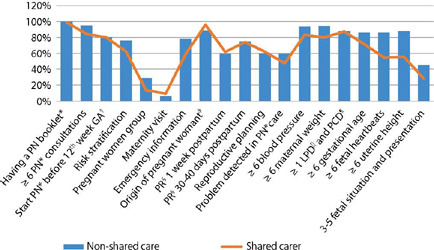
Distribution of interventions to improve use, quality and markers of care according to type of care received, Londrina, Paraná, Brazil, 2022

Interventions for improvements in utilization, quality and markers of care are shown in Figure 2.

*PN – prenatal; †GA – gestational age; ‡Origin of the pregnant woman to carry out childbirth in the same city as the maternity hospital or use of medical transport or ambulance; §PR – puerperal return; ||LPD – last period date; ¶PCD – probable childbirth date.

### Experiencing the difficulty in communication between services

The lack of registration in PNB was evidenced as a typical limitation in the management process, resulting in the lack of communication between services, even though it is considered an essential component to the continuity of shared care:

*We miss most outpatient clinics; it is the counter-referral that does not come; it is an annotation that is not adequate. We also have weakness in this sense with Primary Care, but, for example, it is a note that does not exist in the pregnant women’s card*. (M2)

*Communication between professionals at the reference outpatient clinic and the Primary Care team is important. There are things that we advise that need to be continued.* (M5)

*I refer, on the other hand, my counter-referrence is more or less a poorly filled prenatal card, sometimes with illegible letters, sometimes it just says: exam. Which exam? I don’t know.* (M12)

### Strategies to be carried out for articulation between services

The need for effective counter-referral for continuity of processes and integration between the various points of care in the network was revealed. As strategies evoked by managers in the context of life, professionals’ attitude, financial investment for computerized and unified system implementation, hiring of human resources and the indispensability of guidelines stand out.

[...] *the maternal and child network has been built for many years. We need to continue this construction, continuity of resources, creation of new information systems and hiring of human resources*. (M5)

*There should be a program at the State Department of Health, involving municipal management, secondary and tertiary services, a program that places information in real time* [...] *it would have to have a greater investment so that we could implement and unify this whole process*. (M6)

*I think there is a bit of a lack of explanation from the professional as well. On the mortality committee. I hear it like this, “Pregnant woman in a neonatal death who had not felt the baby move for two days”. I said, “Guys, a mobilogram has to be explained in the first consultation, you know? Because, if she had known that it was serious, that it was not normal, she would have sought care”.* (M9)

*We need to institute this referral and counter-referral process, making professionals aware of the importance of counter-referral*. (M13)

## DISCUSSION

The mixed study made it possible to assess HRPN care, through the identification of the fragility of records in PNB as well as the care performed, in addition to managers’ experience in relation to the management in this segment, revealing gaps and strategies for improvement and continuity of maternal and child care. HRPN care proved to be inadequate in all assessed criteria, regardless of the place where it was carried out. However, shared care showed higher rates, even if they are still low for maternal and fetal assessment, in relation to health system interventions to improve utilization and quality of PN care and care markers.

Corroborated with a study, where the majority of HR pregnant women did not have access to dental care, educational activities and visits to the maternity ward^(16)^.

However, shared care with the SOC proved to be an effective alternative in organizing and improving care quality, even with gaps in its implementation, in terms of nutritional interventions, preventive measures, maternal and fetal assessment, health system interventions to improve utilization and PN care quality and care markers.

For Schütz^(14)^, a subject’s capacity for action occurs naturally in the world of life, in the face-to-face relationship through lived experiences, in which acquired knowledge can be unveiled, in the typical action, from the context of meanings. The study revealed the experiences lived by the team and managers in HRPN care. Knowledge acquired in care actions often falls short, as pointed out by the managers’ reports regarding the fragility of the record in PNB in both services, unveiled in the typical action in the context of meanings to management, weakening communication in the RAS for referral and counter-referral and making it difficult to continue shared care with reciprocity of intentions.

Studies show PNB incompleteness and non-readability, through the absence of records and actions taken. Consequently, this fact generates uncertainty about the actions taken, compromising quality of care^(17, 18)^.

Almost all postpartum women had PNB. The importance of this document is highlighted as a record of medical records, which is a great achievement for quality of care. Also noteworthy are the criteria achieved by PHC and SOC services regarding nutritional interventions regarding the use of supplementation with folic acid and ferrous sulfate, preventive measures for carrying out ABO blood screening tests (classification of human blood), Rh (blood group system) and maternal and fetal assessment for hepatitis B serology, hemoglobin and hematocrit and glucose.

The lack of an institutionalized flow that guarantees the safety of pregnant women when walking between the points of care in the network and the weaknesses in communication between the services and the failure to schedule a subsequent PN consultation can contribute to the non-return of pregnant women to the PHC unit. It is noteworthy that pregnant women, even in HR condition, are the responsibility of the Primary Care team and the Family Health Strategy, and their care should take place at the Basic Health Unit, home visit, among others^(19)^.

Despite the advances already achieved, as well as the *Rede Cegonha* implementation, health actions and services are fragmented and disjointed, in addition to the lack of communication between the points of care, experiencing a context of non-continuity of health actions at the three levels of care, resulting in the disqualification of comprehensive maternal and child care^(15)^.

Each individual is situated in the world of life in a specific way. Considering that, in life, we are faced with elements of possible control and others that are beyond the possibility of control, the subject locates the scene of action, interprets possibilities and faces challenges, this being the biographical situation of man^(14)^. The results of this study pointed to the lack of knowledge of the health team to deal with actions of care and HRPN care management as well as of pregnant women to deal with their health care needs.

In this context, the subject is situated in the world of life, according to the collection or acquisition of knowledge built, considering the experiences lived over time, acquired through their predecessors, and which are interpreted because they have a meaning value, being conveyed by communication, according to what is considered significant, called typification^(14)^.

Since health professionals’ training, it is necessary that their predecessors convey the importance of integration between the different points of care in the RAS so that the importance of interdisciplinarity can be built into each professional’s collection of knowledge, aiming at a reciprocal work, with intentions aimed at a single objective, a single purpose, i.e., resolutive care.

HRPN care follow-up must be carried out through the PHC and SOC networks. It should be noted that the non-guarantee of this care constitutes institutional violence, based on the violation of rights; however, there are challenges to be faced by the municipalities in the reference of SOC, and regulatory systems are still deficient^(19)^.

Faced with the gaps evidenced in HRPN care follow-up, managers revealed expectations (reasons for) regarding the integration between services, which involved professionals’ attitude and the need for a computerized system interconnected with the various points of care to strengthen referral and counter-referral, through financial investment and hiring of human resources.

In Armenia, a study that investigated the main gaps for the electronic health information management system implementation showed the need for financial and human resources for its implementation as well as the support of public policy makers^(20)^.

The computerized system is a valuable resource for managers in the assessment of care. Despite the financial impact of its implementation, the investment can be a strategy for identifying care failures and establishing actions aimed at the identified problems, impacting the maternal-infant mortality indicator.

In this context, the electronic information system implementation must be a global health priority. However, its implementation requires commitment from health leaders to strategic performance in terms of directive policy, resource mobilization and decision-making based on scientific evidence^(21)^.

Awareness and professional commitment to the importance of health education are necessary, which does not require financial investment, depending exclusively on the professional attitude, which is one of the greatest challenges to be faced in care quality.

Caring requires the establishment of a face-to-face relationship, defined as one in which the subjects involved are aware of each other and mutually facing each other in the same time and space^(14)^. In addition to the use of more complex resources, individualized care during HR pregnancy has the strategy of meeting the biopsychosocial needs and ensuring adequate care.

### Study limitations

The study had as a limitation the specific local reality of post-partum women and managers studied, not being possible to generalize in Brazil; however, the data found are representative and can demonstrate the reality of other regions, and can be replicated in other places of interest.

### Contributions to nursing, health, or public policies

This is the first study that classified PN care based on comprehensive care, including criteria for adequacy of care received and the place where it was carried out, contributing to the strengthening of HRPN care actions, pointing out existing gaps as well as possibilities for sharing care. Moreover, the results can serve to redirect public health policies recommended in maternal fetal care.

## FINAL CONSIDERATIONS

HRPN care adequacy, in the municipality under study, showed weaknesses in both PHC and SOC services regarding the incompleteness of records in PNB and non-performance of recommended care. However, when care was shared with the reference service, the quality of care was adequate.

Strengthening the referral and counter-referral was identified as a necessity for the integration and continuity of HRPN care that fundamentally depends on professionals’ attitude regarding the prioritization of care records, the implementation of electronic medical records interconnected with the various levels of care as well as the right of access to health.
